# COVID-19-associated neurological sequelae: A case series on cerebral microbleeds and encephalopathy

**DOI:** 10.5339/qmj.2023.29

**Published:** 2023-11-01

**Authors:** Abeer Sabry Safan, Yahia Imam, Mohamad Y Khatib, Mohammad Al Wraidat, Mohammad M Altermanini, Salah A Al-Mughalles, Anood Al-Assaf, Mariam Olish, Moustafa S Elshafei, Abdulqadir J Nashwan

**Affiliations:** Neurology Department, Hamad Medical Corporation (HMC), Doha, Qatar; Critical Care Department, Hamad Medical Corporation (HMC), Doha, Qatar Email: anashwan@hamad.qa ORCID ID: 0000-0002-0557-6071; Radiology Department, Hamad Medical Corporation (HMC), Doha, Qatar; Department of Internal Medicine, Hamad Medical Corporation (HMC), Doha, Qatar

**Keywords:** COVID-19, SARS-CoV-2, critical illness microbleeds, case series, adult

## Abstract

Background: Critical illness-associated cerebral microbleeds and leukoencephalopathy connected to coronavirus disease 2019 (COVID-19) infection are emerging areas of concern in current medical literature.

Methods: We reviewed cases of patients with COVID-19-associated neurological manifestations to study the prevalence and outcome of such conditions.

Case Series Findings: Our review yielded seven distinct patients. Six exhibited cerebral microbleeds, primarily localized in the juxtacortical white matter and the corpus callosum. In contrast, one individual presented with leukoencephalopathy. Tragically, of these patients, five succumbed to their ailments. One was discharged with mild cognitive impairments, while another underwent a tracheostomy and was subsequently moved to a long-term care establishment.

Conclusion: Our findings underscore the significance of neuro-radiological observations in those grappling with severe manifestations of COVID-19, drawing attention to the possible neurological repercussions of the virus.

## Introduction

Coronavirus disease 2019 (COVID-19) is caused by the severe acute respiratory syndrome coronavirus 2 (SARS-CoV-2) virus.^1^ It was first isolated in Wuhan, China, in December 2019, with rapid and fatal spread worldwide.^2^ As of August 2023, 514,524 cases have been confirmed in Qatar, with 690 deaths.^3^ Varied neurological complications have been described previously with pandemic influenzas, such as facial palsy, anosmia, stroke syndrome, Guillain-Barre syndrome, and rarely acute necrotizing encephalopathy.^4,5^ It is worth noting that neurological manifestation can be the leading cause of presentation, as it is reported in over two-thirds of COVID-19 patients.^6–8^

In a registry of 125 patients with COVID-19, an estimated 39 (31%) presented with altered mental status, of which 23% were diagnosed with unspecified encephalopathy and 18% with encephalitis.^9^ COVID-19-related encephalitis has yet to be fully understood due to data scarcity. Proposed mechanisms vary from a direct viral invasion of the central nervous system (CNS) to an auto-immune molecular mimicry and para- or post-infectious processes.^8^ Ellul et al., in a recent review of neurological associations in COVID-19, estimated that out of globally reported 4–8 million COVID-19 cases, a projected prevalence of up to 9,671 [0.12%] patients with CNS complications, excluding stroke syndromes in patients infected with COVID-19.^10^ Cerebral microbleeds are well-known radiological manifestations of chronic hypertension, cerebral amyloid angiopathy (CAA), diffuse axonal injury, and high-altitude exposure, with variable neuroanatomical predominance.^11,12^ For instance, high-altitude exposure microbleeds commonly involve the corpus callosum, while, in chronic hypertension, they can involve deep gray matter.^11^ On the contrary, critical illness-associated microbleeds are a relatively newly recognized entity in critically ill patients with severe respiratory failure.^12,13^

## Methods

In this case series, we reported COVID-19 patients from Hazm Mebaireek General Hospital (HMGH), Qatar's main COVID-19 designated facility, who exhibited distinct neuroimaging findings from December 2019 to December 2021. Patients were included if they were diagnosed with COVID-19 as per the World Health Organization (WHO) definition^3^ and had undergone magnetic resonance imaging (MRI) scans showing microbleeds in the juxtacortical white matter and corpus callosum or signs of leukoencephalopathy. Our categorization of infectious encephalitis was based on the criteria from Wu et al.,^14^ and the clinical classification followed the WHO's guidelines for coronavirus infections. Clinical data from electronic medical records covered demographics, clinical presentation, lab findings, and neuroimaging results. MRI scans were evaluated by board-certified radiologists specializing in neuroimaging, with a consensus approach to resolving discrepancies. Our analysis primarily employed descriptive statistics to detail the clinical and neuroimaging characteristics of the patients. The probability of COVID-19-related neurological complications was based on a recently published review by Ellul et al.^10^ and is illustrated in Table 1.

### Case I

**Figure 1.**
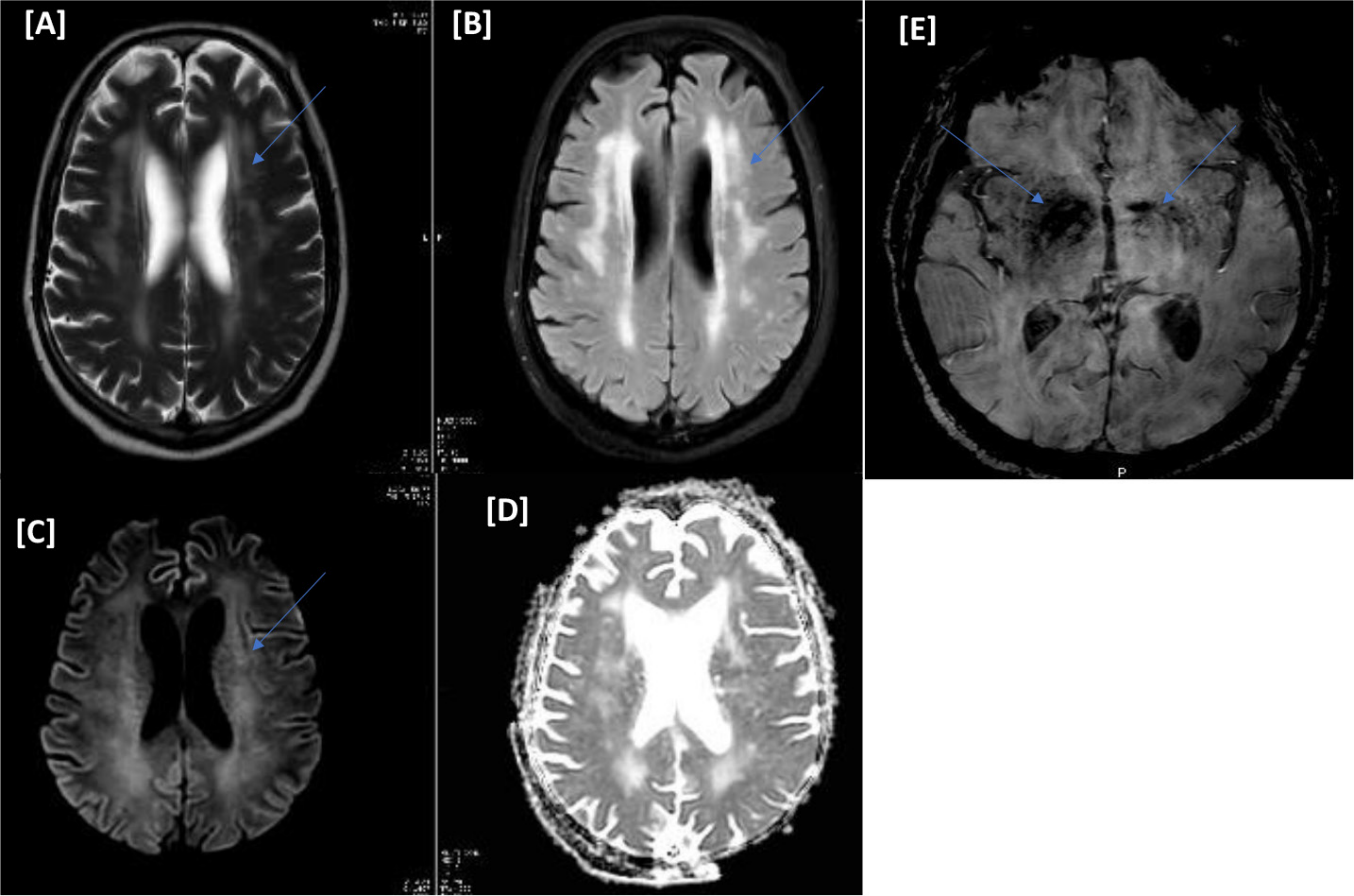
(Case I) [A–E images] Magnetic resonance imaging of the head. Diffuse periventricular white matter hyperintense signals on T2/FLAIR (A and B). No corresponding diffusion restriction on diffusion-weighted imaging b1000/apparent diffusion coefficient (C and D). Bilateral basal ganglia small storm susceptibility appearance on susceptibility-weighted images is more significant on the right side (E).

A 70-year-old male with a medical history of diabetes mellitus (DM), hypertension, and multiple myeloma (in remission phase) presented to the accident and emergency (A&E) department with respiratory symptoms of cough, shortness of breath, and fever. Nasopharyngeal swab polymerase chain reaction (PCR) was positive for COVID-19. His condition deteriorated, requiring ventilatory support and medical intensive care unit (MICU) admission. Two days after the admission, the patient was intubated. He was treated in line with the COVID-19 local protocol (hydroxychloroquine, dexamethasone, and antibiotics). He was tracheostomized after 14 days due to difficulty weaning off ventilation. Despite sedation cessation, his level of consciousness did not improve, warranting further neuroimaging. MRI of the head showed extensive brain parenchyma periventricular diffuse white matter with increased T2-signal intensity. Susceptibility-weighted images (SWI) showed subcortical microbleeds of the brainstem and the basal ganglia (Figure 1AߝD). A lumbar puncture showed no pleocytosis, with negative viral panel and cultures. COVID-19-related encephalopathy was suspected. He was treated with two doses of tocilizumab followed by convalescent plasma therapy; however, the patient did not improve. Laboratory investigations showed severe hyperkalemia requiring dialysis due to the failure of the initial medical measures. He spent 74 days in the MICU, which was complicated by refractory hyperkalemia despite dialysis; this culminated in cardiac arrest and death (see Table 2).

### Case II

A 48-year-old female with a past medical history of longstanding DM and hypertension presented to the A&E department with a 10-day history of fever, sore throat, and myalgia. COVID-19 PCR from a nasopharyngeal swab was positive. The chest X-ray showed bilateral infiltrates. MICU admission was necessary due to increased respiratory effort and oxygen demand; the patient required intubation and mechanical ventilation for 15 days. She was successfully extubated on day 15. On day 17, her level of consciousness deteriorated, warranting further medical investigations. No other metabolic derangement was uncovered.

**Figure 2.**
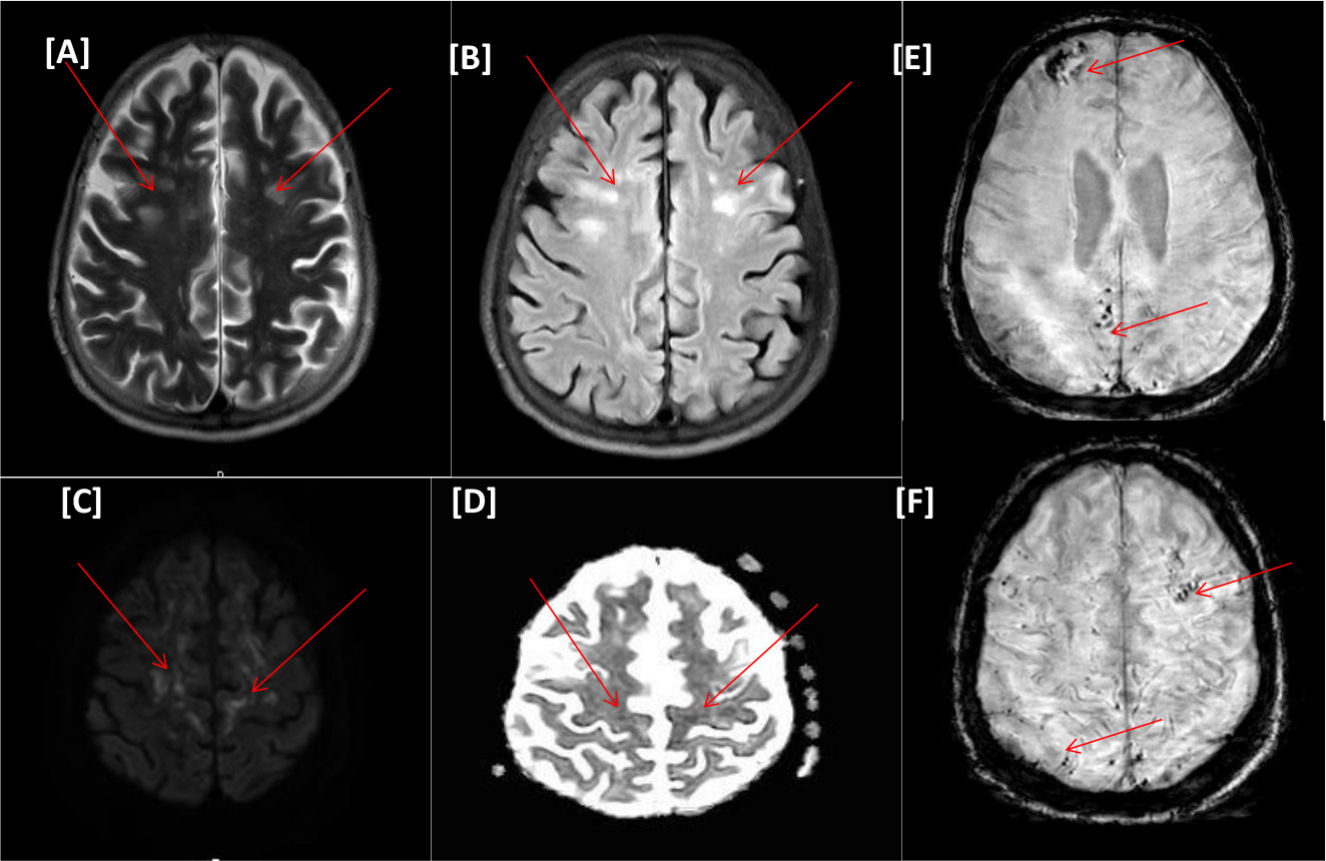
(Case II) [A–F images] Magnetic resonance imaging of the head. Bilateral scattered multifocal variable-sized nodular and punctate areas of hyperintense signals on T2WI/FLAIR (A and B). Foci of diffusion restrictions on diffusion-weighted imaging series b1000/apparent diffusion coefficient (C and D), denoting acute ischemic changes. Foci of susceptibility artifacts on susceptibility-weighted images, microhemorrhagic changes (E and F).

The MRI of the head (Figure 2) showed faint bright signal intensity on the diffusion-weighted imaging (DWI), with a corresponding iso-signal on the apparent diffusion coefficient (ADC) and bright signal intensity on the T2/FLAIR sequence involving the precentral subcortical white matter; features were suggestive of encephalitis. Microbleeds were noted on the SWI sequence and were of variable size, involving the splenium of the corpus callosum. The clinical and radiological findings suggested possible COVID-19 encephalitis/critical illness-related microbleeds. The patient received two doses of tocilizumab (400 mg and 600 mg). Her level of consciousness gradually improved, and she continued to recover with intensive rehabilitation, addressing cognitive and critical illness neuromyopathy. The overall condition improved, and she was discharged with minimal assistance in walking with regular outpatient follow-up appointments (see Table 2).

### Case III

A 57-year-old male with no known medical history presented to the A&E department with respiratory symptoms and fever; subsequently, COVID-19 PCR from the nasopharyngeal swab was positive. The patient developed respiratory distress, warranting MICU admission and intubation. Due to the failure of weaning off ventilation, he was tracheostomized, totaling 56 days of intensive care stay. Glasgow Coma Scale (GCS) was 2T despite cessation of sedation, raising the suspicion of COVID-19-related encephalopathy, mainly because no other metabolic derangement was uncovered.

**Figure 3.**
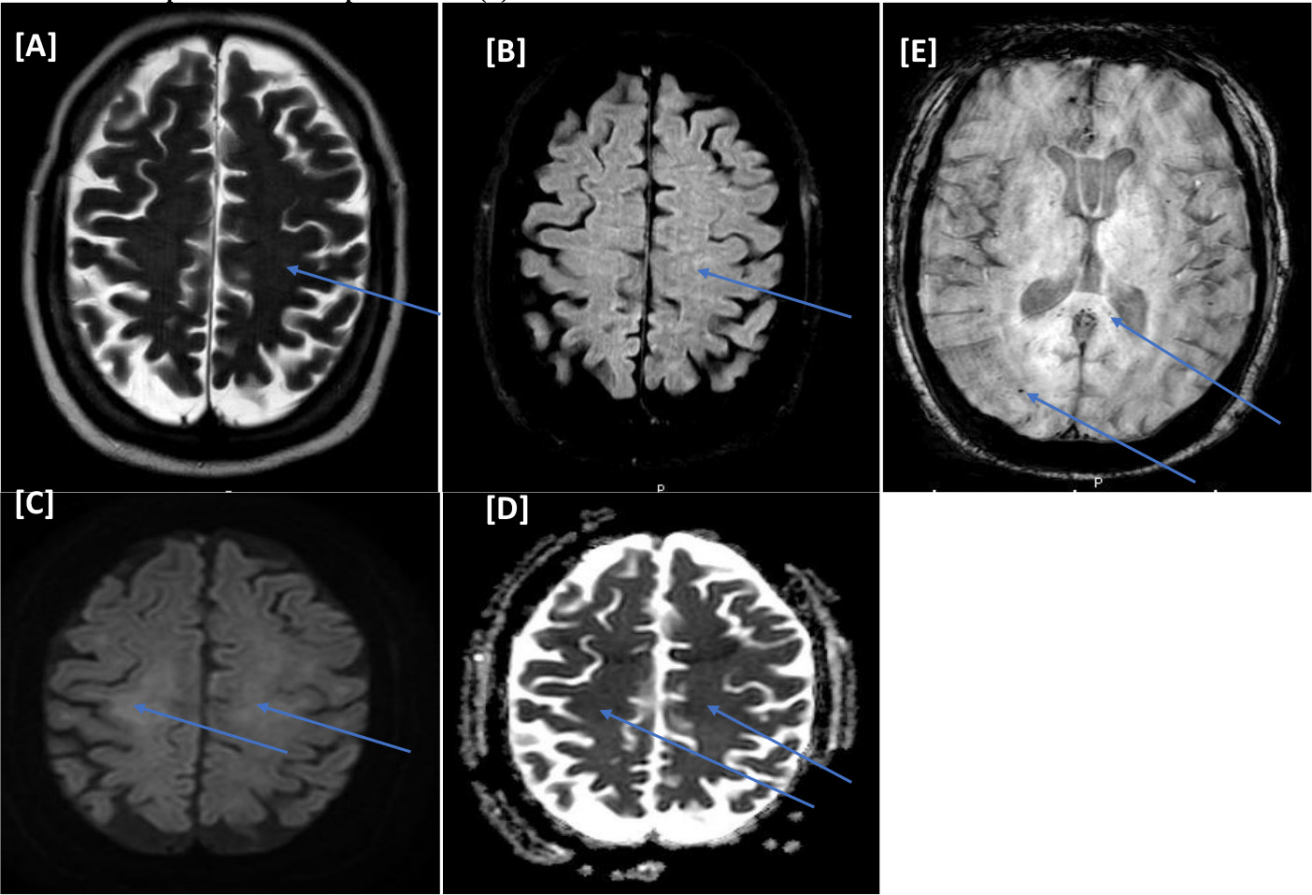
(Case III) [A–E images] Magnetic resonance imaging of the head. Bilateral centra semiovale white matter faint bright signal intensities on T1WI/FLAIR (A and B) with corresponding faint restriction on diffusion-weighted imaging series b1000/apparent diffusion coefficient (C and D). There are numerous variable-sized foci of blooming artifacts (microbleeds) on susceptibility-weighted images at the subcortical and splenium of the corpus callosum (E).

An MRI of the head (Figure 3) showed microbleeds predominately in the splenium of the corpus callosum. He received convalescent plasma. Unfortunately, the MICU course was complicated with septic shock and disseminated intravascular coagulation (DIC) with multi-organ failure. Despite maximum vasopressor support, the patient developed pulseless electrical activity and passed away on day 63 of his MICU stay (see Table 2).

### Case IV

**Figure 4.**
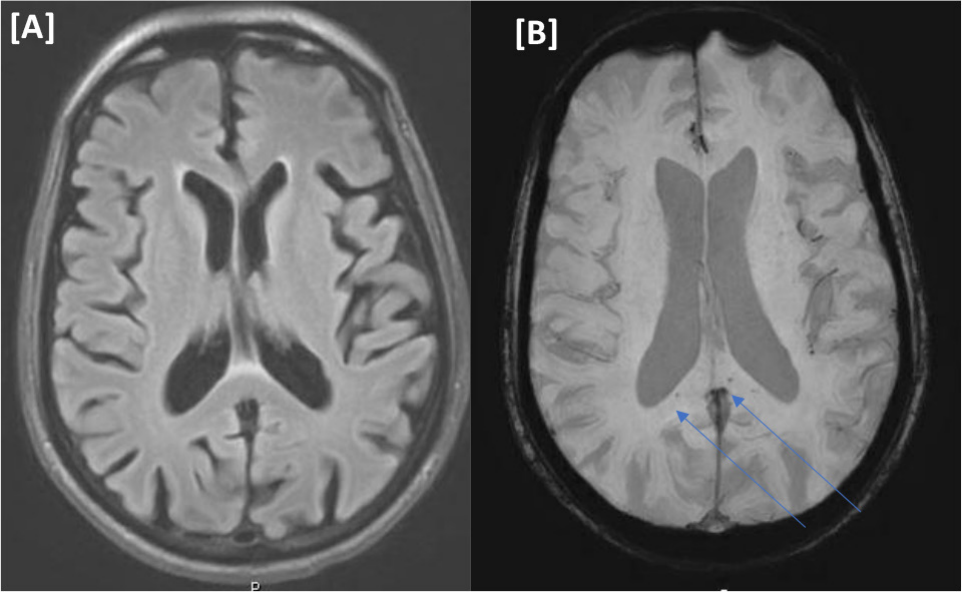
(Case IV) [A and B images] Magnetic resonance imaging of the head. No focal parenchymal abnormal signal intensity on FLAIR (A). Few punctate foci of microbleeds at the splenium of the corpus callosum on susceptibility-weighted images in panel (B).

A 74-year-old male presented to the A&E department with respiratory symptoms of cough, shortness of breath, and fever. COVID-19 infection was confirmed via a nasopharyngeal swab. The next day, his condition deteriorated, requiring ventilatory support. Due to his impaired level of consciousness despite cessation of sedation, he was not weaned off ventilation and was eventually tracheostomized. Further clinical investigations revealed an MRI of the head with multiple acute lacunar infarcts in the right deep frontal region with diffusion restriction. SWI sequence showed numerous microbleeds with corpus callosum and deep gray matter consistent with possible overlapping findings of COVID-19 encephalopathy with critical illness cerebral microbleeds (Figure 4AߝD). The patient received one dose of convalescent plasma. The MICU course spanned over 95 days and was complicated by fungemia, and refractory septic shock with multi-organ failure, culminating in cardiac arrest (asystole) and death (see Table 2).

### Case V

**Figure 5.**
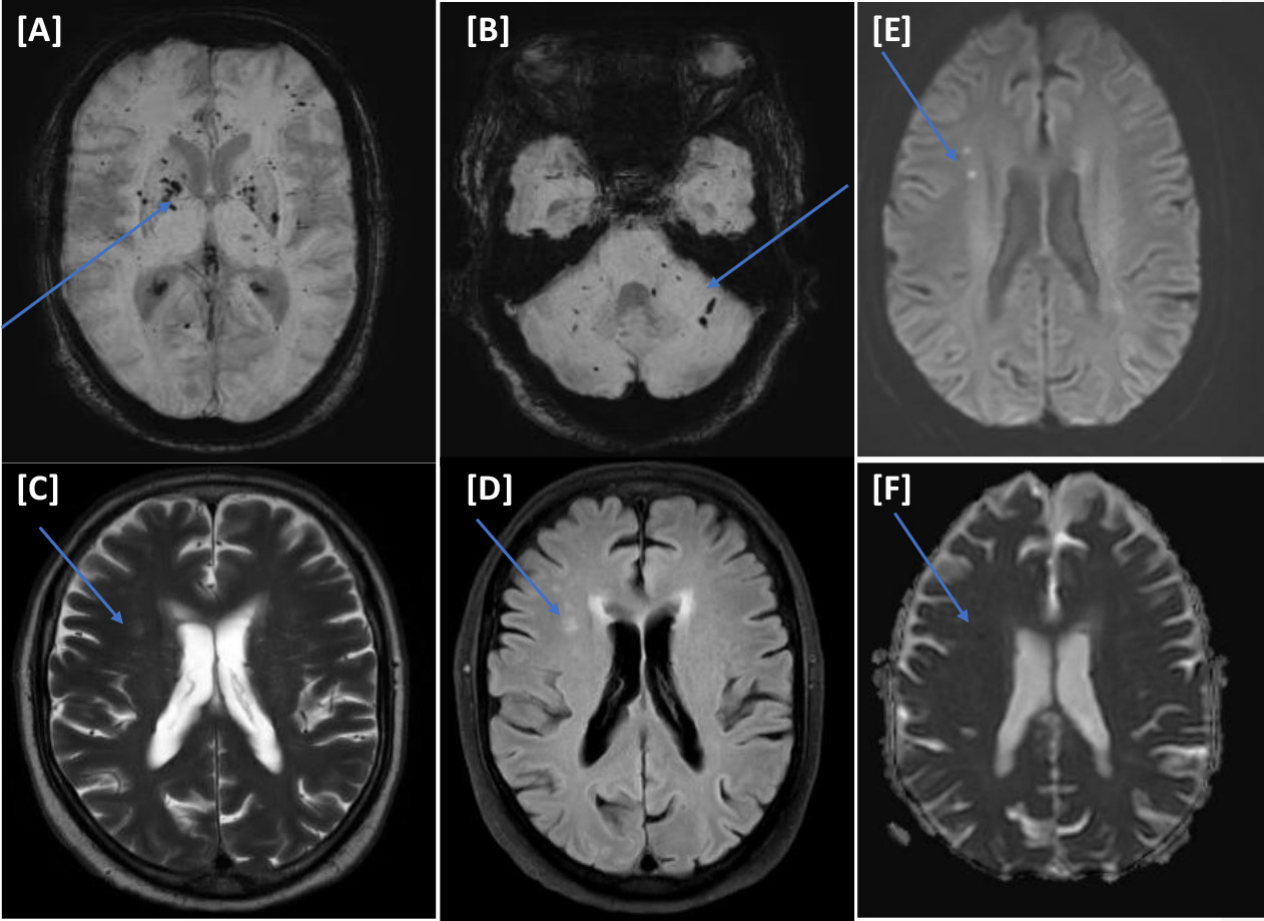
(Case V) [A–F images] Magnetic resonance imaging of the head. Widely scattered variable-sized tiny foci of blooming artifacts (microbleeds) on susceptibility-weighted images at supratentorial (A) and infratentorial levels (B). Foci of bright signal intensities on T2WI/FLAIR (C and D) in the right frontal deep white matter with corresponding diffusion restrictions on diffusion-weighted imaging series b1000/apparent diffusion coefficient (E and F), representing acute infarctions.

A 64-year-old male with no significant past medical history presented to the A&E department with respiratory failure due to COVID-19 pneumonia, warranting MICU admission. The patient was intubated and subsequently tracheostomized on day 15. Laboratory studies showed a low platelet count (with a nadir of 31,000 per microliter) and positive heparin-induced thrombocytopenia studies. Throughout his MICU course, his level of consciousness failed to improve. The MRI of the head showed features suggestive of COVID-19-encephalopathy with microbleeds involving the corpus callosum, the subcortical and deep white matter, and the gray matter structures (Figure 5). No immunotherapy was utilized. His course was complicated by bilateral pneumothorax, acute kidney injury requiring renal replacement therapy, and secondary infection with resistant bacteremia. The patient's condition ultimately deteriorated; brainstem reflexes were absent, and he was declared brainstem dead after the 15th day of admission (see Table 2).

### Case VI

**Figure 6.**
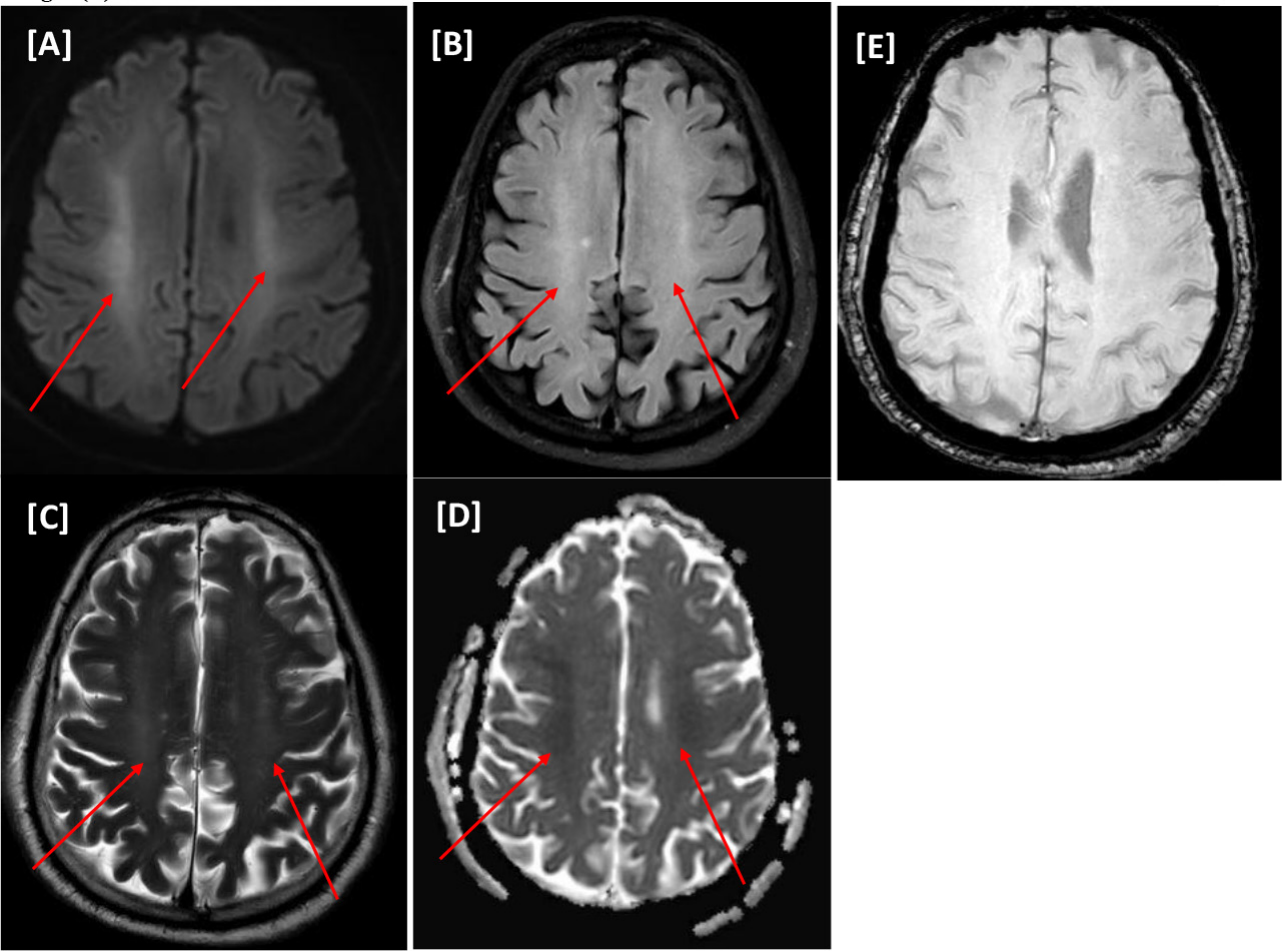
(Case VI) [A–E images] Magnetic resonance imaging of the head. Patchy rather symmetrical areas of bright signal intensities on T2WI/FLAIR involving the white matter of bilateral centra semiovale (A and B) with corresponding mild restriction patterns on diffusion-weighted imaging series of faint bright signals on b1000 and low value on apparent diffusion coefficient map (C and D). No microhemorrhage on susceptibility-weighted images (E).

A 66-year-old male with a medical history of DM, coronary artery disease, hypertension, and chronic kidney disease (CKD) presented to the A&E department with progressive respiratory symptoms. He was found to have a positive COVID-19-PCR nasopharyngeal swab. He was initially admitted to the inpatient unit for two days and then transferred to MICU by the Rapid Response Team (RRT) due to a deterioration in consciousness level requiring intubation. On day 21 of MICU admission, an MRI of the head showed bilateral symmetrical frontoparietal subcortical and deep white matter changes suggestive of COVID-19-related leukoencephalopathy; no microbleeds were present on the SWI sequence (Figure 6).

He received two doses of convalescent plasma. His hospital stay was further complicated by an acute kidney injury requiring renal replacement therapy and secondary infection with extensively drug-resistant organisms. Despite gradual improvement in consciousness, he was tracheostomized due to weaning failure after approximately one month of his MICU stay. He was transferred to a long-term care facility for further pulmonary and physical rehabilitation for severe critical illness myopathy (see Table 2).

### Case VII

A 67-year-old male with a distant past medical history of Hodgkin's lymphoma in remission, hypertension, DM, CKD, and coronary artery disease presented to the A&E department with fever and severe shortness of breath and was diagnosed with COVID-19 pneumonia. He was admitted to the MICU and intubated ten days later due to increased oxygen requirement.

During the hospital course, he developed septic shock with multi-organ failures and DIC requiring inotropes and renal replacement therapy (RRT). The patient was referred to nephrology for acute CKD following a significant elevation in creatinine levels from a baseline of around 150. The creatinine measurements showed a fluctuating upward trend: 187, 168, 152, 137, 148, 157, 298, 344, and 513 μmol/L. On November 11, 2020, the patient experienced a deterioration of health condition, marked by tachycardia, hypotension (blood pressure 75/50 mmHg), and fever. At that time, the patient was on 3 μg of phenylephrine. Laboratory tests revealed line-related Gram-negative bacteremia caused by *Klebsiella pneumonia* on the same day, following which the patient was put on intravenous antibiotics. The deterioration of renal function commenced on the same day, likely due to acute tubular necrosis (ATN) secondary to septic shock. The first hemodialysis session was initiated two days later in response to the worsening renal condition.

**Figure 7.**
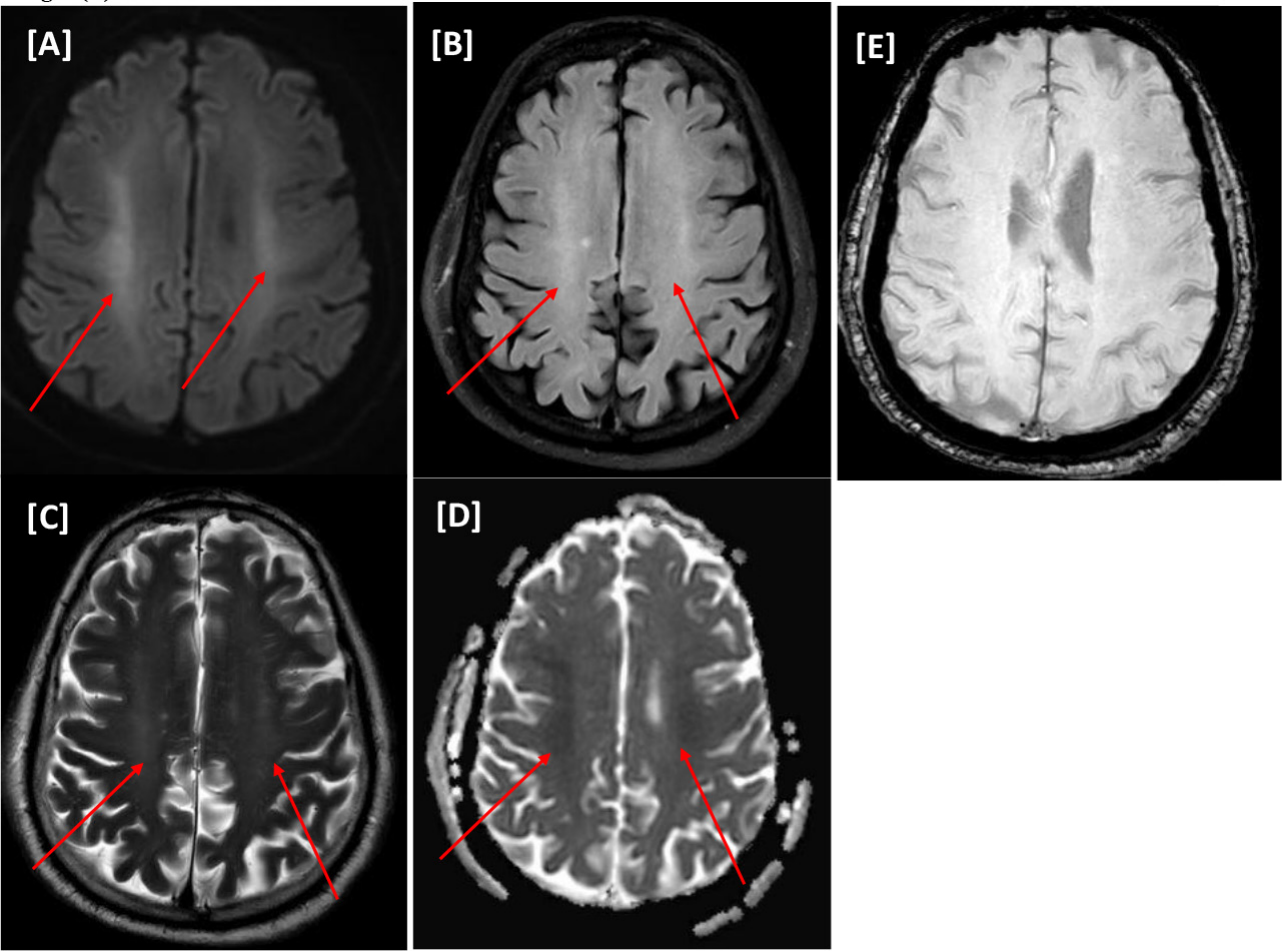
(Case VII) [A–E images] Magnetic resonance imaging of the head. Bilateral almost symmetrical focal subcortical high frontal areas of hyperintensities foci on T2WI/FLAIR sequences (A and B) with corresponding diffusion restriction on diffusion-weighted imaging series b1000/apparent diffusion coefficient (C and D). No microhemorrhage on susceptibility-weighted images (E).

A head MRI was performed nearly 30 days after admission to the MICU. It revealed multifocal parenchymal hemorrhagic foci on the SWI sequence, displaying findings that overlapped with those typically associated with critical illness and COVID-19 microbleeds (Figure 7). The patient's condition deteriorated, leading to his demise 35 days post-MICU admission (Table 2).

## Discussion

We report seven cases of severe COVID-19 pneumonia requiring intubation and intensive case support. All the patients had an impaired level of consciousness, warranting further neuroimaging. Five patients had radiological features suggestive of critical illness-associated microbleeds, one had microbleeds in deep gray matter, cerebellum, and corpus callosum suggestive of overlapping etiology, and one had extensive white matter changes suggestive with COVID-19-associated leukoencephalopathy and no microbleeds (Case VI).

According to Ellul et al., this case series shows probable (two cases) or possible (five cases) COVID-19 encephalopathy, with radiological overlap with possible critical illness microbleeds.^10^ COVID-19-related encephalopathy radiological patterns vary from leptomeningeal enhancement, ischemic strokes, and high T2 signal in the cortex.^4^

Patients admitted to the intensive care unit (ICU) commonly have multifactorial factors for decreased levels of consciousness, including hypoperfusion, infection, and multiorgan failure.^11^ Unentangling COVID-19's contribution to critical illness microbleeds is difficult with MRI scanning, which plays a prominent role in helping rule out confounding causes and planning management strategies and prognostication. Critical illness-associated microbleeds on SWI MRI sequences are uncommon and poorly understood findings.^13,15,16^

Toback et al. demonstrated in a recent study of 279 patients with critical illness microbleeds that two cases showed a distinctive pattern of distribution involving the juxtacortical white matter and callosal region, sparing the deep and periventricular white matter, basal ganglia, and thalami, as observed in our reported Cases II–IV.^17^ The pathophysiology of such a unique phenomenon is not fully understood, but the proposed mechanism is attributed to multiple systemic factors (hypoxemic, hydrostatic, and coagulopathy).^17–19^

Fanou et al. described frequent corpus callosal involvement microbleeds in a case series hypoxemic due to high-altitude exposure or critical illness.^11^ Proposed mechanisms include hypoxemia-induced hydrostatic and chemical effects on the blood–brain barrier, resulting in extravasation of erythrocytes, brain endothelial erythrophagocytosis, and oxidative stress across the brain endothelium.^11,13^ However, cerebral microbleeds have been noted in cases of coagulopathy, as seen in conditions such as thrombotic thrombocytopenic purpura (TTP), Idiopathic thrombocytopenic purpura (ITP), and hematological malignancies. A recent study highlighted this, showing that 43% of ITP patients (21 out of 49) exhibited microbleeds in various regions, such as the cortex, subcortex, and corpus callosum, suggesting a link to thrombocytopenia.^20^ Despite Cases I and VII having a history of multiple myeloma and Hodgkin's lymphoma, respectively, they were both in remission with no previous reported neurological sequela or MRI of the head to identify background cerebral microbleeds.

Intriguingly, corpus callosum involvement is often observed in our case series of critically ill COVID-19 patients; however, the cofounding impact of other vascular risk factors or possible undiagnosed early CAA can explain the overlapping neuroanatomical locations of microbleeds.^13^ To date, management of critical illness microbleeds remains supportive, with addressing the possible underlying etiology being the mainstay treatment proposed in the literature.^13,15^

COVID-19-related encephalopathy outcomes exhibit variability, and several factors can contribute to its occurrence.^21^ According to a comprehensive prospective study conducted in 2023 involving over 4000 COVID-19 patients, 9.2% experienced acute encephalopathy.^21^ The multivariable analysis performed during the 90-day follow-up of this study revealed that individuals older than 70 years, who required RRT while in the ICU, and those who developed CNS ischemic or hemorrhagic complications, had a poor outcome due to acute encephalopathy, with odds ratios (ORs) estimated at 4.01, 2.31, and 3.22, respectively.^21^ In our series of cases, six patients developed CNS hemorrhagic complications in microbleeds. Cases III, IV, and VII required inotropes, and Cases I, V, VI, and VII underwent RRT, explaining the unfavorable outcomes. Among our patients, five succumbed to the condition, one was discharged home with mild cognitive impairment, and one experienced a tracheostomy and was transferred to a long-term care facility.

Most of our reported cases fall in the possible category of COVID-19-related neurological manifestations, as per Ellul et al., owing to their complicated course with critical multisystem involvement such as sepsis and electrolyte derangement and coagulopathy (disseminated intravascular coagulopathy).^10^ Despite the lack of consensus on treating COVID-19 patients; the commonly used medication is interleukin-6 (IL-6) receptor antagonist (tocilizumab).^22^ In our case series, two (Cases I and II) out of the seven patients were treated with tocilizumab (TCZ), with one patient (Case II) having a favorable outcome. In a retrospective cohort study, 515 patients received TCZ and steroids, with an estimated 73.8% having clinical improvement. In comparison, some data support TCZ use in COVID-19-related multisystem complications, such as cytokine release syndrome.^22^ This data originates from multiple retrospective studies with an overarching aim of reducing the risk of invasive ventilation and, ultimately, death. The impact of such a regimen on COVID-19-associated encephalopathy is unclear.^23^ Muccioli et al. reported a case of COVID-19 encephalopathy presenting with frontal lobe dysfunction and aphasia that resolved with a TCZ regimen; however, limited data are available to date to discern its usefulness in COVID-19-associated encephalopathy.^23^ Furthermore, Meshref and colleagues have reported four cases of cerebrovascular events in COVID-19 patients, highlighting the neurological complications of the virus.^12^ The cases include a 33-year-old man with a cerebellar infarct, a 58-year-old man with acute ischemic stroke, a 40-year-old woman with cerebral venous sinus thrombosis, and a 64-year-old man with acute hemorrhagic stroke. These cases emphasize the need for increased vigilance and prompt management of cerebrovascular complications in COVID-19 patients. Understanding the pathophysiology and risk factors associated with these events can help improve patient outcomes and guide future research in this area. While management of microbleeds includes correcting underlying coagulopathy in cases of hematological diseases such as ITP, management in critical illness microbleeds without clear quantitative coagulopathy remains supportive, with more research needed to understand its implications and sequela in COVID-19 and critically ill patients and whether immunotherapy could help alleviate or prevent it.

## Conclusion

We present a case series involving six COVID-19 patients who exhibited microbleeds in the juxtacortical white matter and the corpus callosum and a separate case of leukoencephalopathy. The co-occurrence of COVID-19 encephalopathy and critical illness-associated cerebral microbleeds is a complex clinical phenomenon that poses significant challenges for medical practitioners seeking to disentangle their interconnectedness. COVID-19 encephalopathy refers to the manifestation of various neurological symptoms such as confusion, impaired consciousness, and cognitive disturbances exhibited by some patients with COVID-19. On the contrary, critical illness-associated cerebral microbleeds are small cerebral hemorrhages often found in severely ill patients and can indicate vascular damage. Although this combined occurrence is well-documented, the clinical implications of neuroimaging findings, in which both conditions are evident, remain nebulous and represent a vibrant field of research. Detecting these conditions through neuroimaging could potentially serve as a prognostic marker for the severity of the disease. This hypothesis stems from the observation that such findings are predominantly reported in critically ill patients, who frequently present with many confounding factors, including vascular complications and systemic disorders. However, it is essential to note that the degree to which these conditions indicate disease severity is still under investigation, and further research is warranted to understand their clinical significance more fully. The next step in advancing our knowledge would be to conduct comprehensive studies to elucidate the frequency and patterns of this co-occurrence. This would provide essential insights into the prevalence and nature of these findings in the patient population affected by severe COVID-19 infection.

## Ethics approval and consent to participate

The study adhered to the Helsinki Declaration and was approved by the Medical Research Center at Hamad Medical Corporation [MRC-04-21-550].

## Consent for publication

Written informed consent was obtained from the patient's next of kin to publish the details of their medical care and any accompanying images.

## Availability of data and material

All data generated or analyzed during this study are included in this published article.

## Competing interests

The authors declare that they have no competing interests.

## Authors contributions

AS and MK were involved in the manuscript's conception, design, and drafting. MAW, YI, MMA, SA-M, AA-A, MO, ME, and AN were involved in the data collection, revising it critically for intellectual content and the final approval of the version to be published. All authors agree to be accountable for all aspects of the work.

## Acknowledgments

We want to acknowledge and honor the significant contributions of our late co-author, Dr. Anood, who sadly passed away on April 25, 2023. Dr. Anood's dedication, expertise, and insights greatly enriched this work, and we are grateful for the opportunity to have collaborated with such a talented researcher. Our thoughts are with Dr. Anood's family, friends, and colleagues during this difficult time.

## Figures and Tables

**Table 1.** Definition of COVID-19-related neurological complications based on WHO and Ellul et al.10

SARS-CoV-2 meningitis, encephalitis, myelitis, or CNS vasculitis*	
Confirmed	SARS-CoV-2 detected in CSF or brain tissue or evidence of SARS-CoV-2-specific intrathecal antibody No other explanatory pathogen or cause found
Probable	SARS-CoV-2 detected in respiratory or another non-CNS sample or evidence of SARS-CoV-2-specific antibody in serum indicating acute infection. No other explanatory pathogen or cause found
Possible	Patient meets the suspected case definition of COVID-19 according to national or WHO guidance based on clinical symptoms and epidemiological risk factors; in the context of known community SARS-CoV-2 transmission, supportive features include the following: the new onset of at least one cough, fever, muscle aches, loss of smell, or loss of taste; lymphopenia or raised D-dimer level; and radiological evidence of abnormalities consistent with infection or inflammation (e.g., ground glass changes)
**Acute disseminated encephalomyelitis associated with SARS-CoV-2 infection, Guillain-Barré syndrome, and other acute neuropathies associated with SARS-CoV-2 infection.**	
Probable association	Neurological disease onset within six weeks of acute infection Either SARS-CoV-2 RNA detected in any sample or antibody evidence of acute SARS-CoV-2 infection No evidence of other commonly associated causes
Possible association	Neurological disease onset within six weeks of acute infection Either SARS-CoV-2 RNA detected in any sample or antibody evidence of acute SARS-CoV-2 infection Evidence of other commonly associated causes

Abbreviations: SARS-CoV-2, severe acute respiratory syndrome coronavirus 2; COVID-19, coronavirus disease 2019; WHO, World Health Organization.

**Table 2.** Characteristics of the reported cases.

	**Case I**	**Case II**	**Case III**	**Case IV**	**Case V**	**Case VI**	**Case VII**
Age (years)	70	48	57	74	64	66	67
Sex	M	F	M	M	M	M	M
Past medical history/comorbidities	DM2, HTN, MM	DM2, HTN	DM2	None	None	DM2, HTN, CAD, CKD	DM2, HTN, CAD, CKD, OSA
COVID-19 symptoms	Fever, dry cough, SOB, with myalgia	Fever, cough with sputum, generalized body ache, diffuse abdominal pain, running nose, and SOB	Fever and SOB	Fever, SOB, tongue pain/ulceration, and dry cough	Cough, SOB, fever	Fever and runny nose	SOB
Supplemental oxygen	Yes	Yes	Yes	Yes	Yes	Yes	Yes
Mechanical ventilation	Yes	Yes	Yes	Yes	Yes	Yes	Yes
Delay between COVID-19 onset and neurological symptoms (days)	31	13	46	54	40	25	26
Neurological symptoms	Low GCS	Low GCS	Low GCS	Low GCS	Low GCS	Low GCS	Low GCS
Cellularity, cells/mm3	1	N/A	N/A	1	N/A	N/A	N/A
Protein levels, g/L	0.3	N/A	N/A	0.47	N/A	N/A	N/A
SARS-CoV-2 RT-PCR assay	Positive	Positive	Positive	Positive	Positive	Positive	Positive
Encephalopathy classification	Probable	Probable	Possible	Possible	Possible	Possible	Possible

Abbreviations: M: male; F: female; DM2: diabetes mellitus type 2; HTN: hypertension; MM: multiple myeloma; SARS-CoV-2: severe acute respiratory syndrome coronavirus 2; RT-PCR: reverse transcription-polymerase chain reaction; COVID-19: coronavirus disease 2019; GCS: Glasgow Coma Scale; CAD: coronary artery disease; CKD: chronic kidney disease; OSA: obstructive sleep apnea; SOB: shortness of breath; N/A: not available.
